# Screening for ROP

**Published:** 2017

**Authors:** Graham Quinn, Clare Gilbert

**Affiliations:** Professor Emeritus of Ophthalmology: The Children's Hospital of Philadelphia, Wood Center, Philadelphia, USA.; Professor of International Eye Health and Co-director: International Centre for Eye Health, London School of Hygiene & Tropical Medicine, London, UK.

**Figure:**
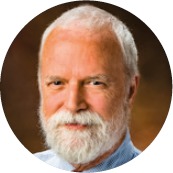
Graham Quinn

**Figure:**
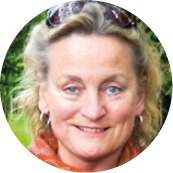
Clare Gilbert

**Screening babies for ROP is very important. Unless ROP is detected early and promptly treated, it can lead to blindness and permanent visual impairment. This article describes who to screen, when and where to screen, how to screen, and what to do next.**

## Why is screening needed?

Treatment for severe ROP is usually successful in preserving vision as long as treatment is given on time by an experienced ophthalmologist. The purpose of screening is to identify babies who need urgent treatment.

## How and where should screening be done?

**Figure 1:**
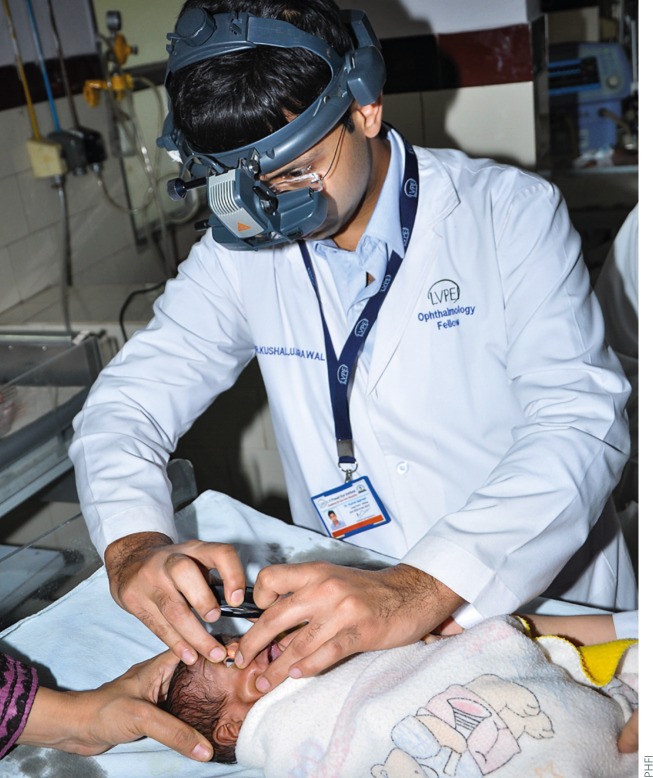
Ophthalmologist screening for ROP using indirect ophthalmoscopy

Most screening for ROP is undertaken by an ophthalmologist, using indirect ophthalmoscopy (Figure 1).

Babies who are in-patients in the neonatal unit must be screened in the unit. Babies who need further screening after discharge can be bought back to the unit for screening or they can be screened in the eye department.

**Figure 2:**
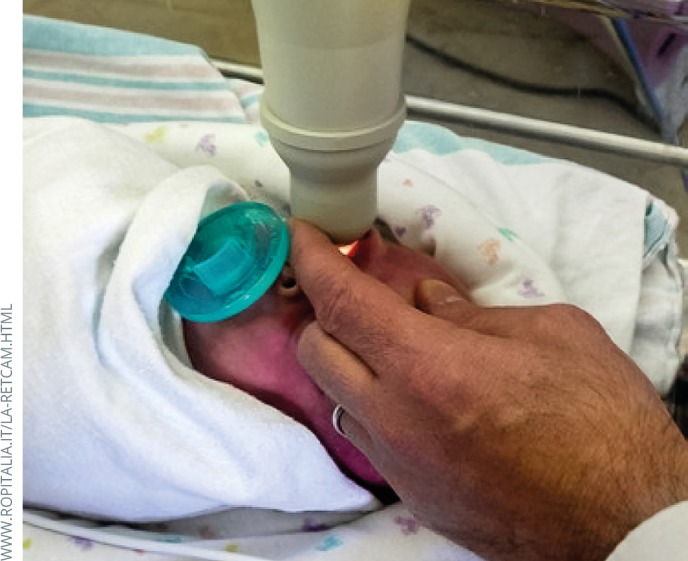
Screening using a RetCam, which uses a probe placed gently on the eye

Over the last few years, wide-field digital imaging systems, instead of indirect ophthalmoscopy, have also been used for screening. The retinal image can be captured by an ophthalmologist, a trained nurse, or a technician (Figure 2). However, an experienced ophthalmologist must always be available to interpret the images.

The screening results for each eye must be classified according to the criteria set up by the International Committee for the Classification of ROP (see pp. 55–56).

## Which babies should be screened?

This is an important question. Which babies are at risk of severe ROP varies considerably. For example, in units where neonatal care is less than ideal, bigger, more mature babies can still develop severe ROP.

Several countries have national guidelines indicating which babies should be screened. These usually include a combination of birth weight (BW) and gestational age (GA). Some countries, such as the United States of America, include additional ‘sickness criteria’ alongside BW and GA. In neonatal units providing very high quality care, only the most preterm babies are at risk of developing ROP and therefore need to be screened.

- In the United Kingdom, babies with BW of <1,250 g, or a GA of 31 weeks or less, must be screened.
- In the United States of America, the screening criteria are a BW of 1,500 g or less, or a GA of 30 weeks or less. Infants with a BW between 1,500 g and 2,000 g should also be screened if they have had an ‘unstable clinical course.’
- In China, a middle-income country, the criteria are BW <2,000 g or GA <34 weeks. Compared with the UK and USA, older and bigger babies in China are considered to be at risk of developing ROP.

**Figure 3:**
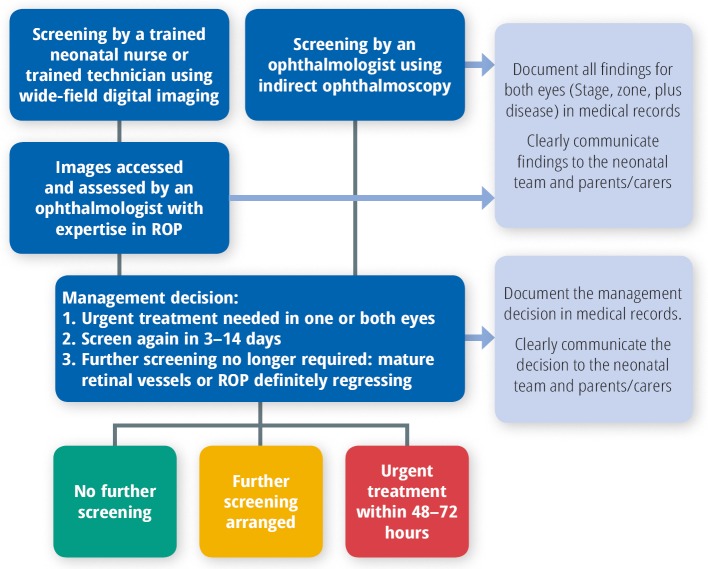
Screening options for ROP and the information to be documented and communicated

Ideally, studies need to be done in each country to determine which babies should be included in a screening programme.

Whichever criteria are used, it is the responsibility of the neonatologist to identify which babies should be screened, and a neonatal nurse should prepare the babies for screening (p. 54).

## When screening should start

Preterm babies are not born with ROP; it only develops during the first few weeks after birth.

It is useful to have guidelines for the timing of the first screening which are easy to implement, particularly in settings where information on GA is unreliable.

**Figure 4:**
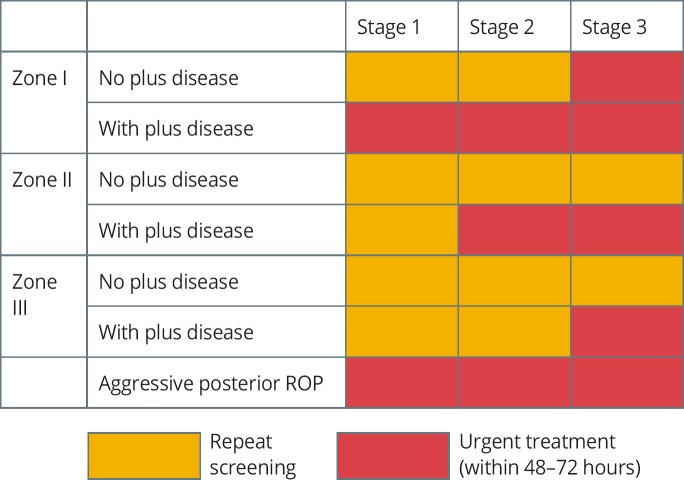
Indications for urgent treatment from the Early Treatment of ROP Trial

For example, screen by 30 days of life. If the baby is very premature, or has been very sick or received a lot of oxygen, earlier screening should be considered. Current thinking suggests screening between 21 and 25 days of life, but more research is needed. If a baby eligible for screening is to be discharged or transferred to another neonatal unit before the first screening, they should be screened before discharge or transfer.

## Understanding the findings of screening

- In eyes where the retinal blood vessels can only be seen in zone I at the first screening, about half will go on to develop ROP needing treatment.
- If the retinal blood vessels have reached zone II at the first screening, ROP needing treatment is unlikely.
- If mature vessels can be seen in zone III, ROP needing treatment is rare.

## Making decisions

At each examination, a management decision needs to be made, based on the eye with the most advanced ROP (Figure 3).

The possible management decisions are:

1. Urgent treatment.
2. Further screening is needed (see below).
3. No further screening is needed as the retinal blood vessels are mature, or ROP is regressing in both eyes.

If urgent treatment is needed, this must be delivered within 48 to 72 hours. If further screening is needed, the date of the next screening examination must be documented and explained to parents. Figure 4 shows which babies need urgent treatment.

## Repeat screening

Findings at the first examination determine when the next screening should take place.

- If the retinal vessels are immature and there is no ROP, follow-up screening can be conducted 1–2 weeks later.
- If there is Stage 1 ROP in zone II with no plus disease, repeat screening in 1 week.
- If there is Stage 2 ROP in zone II with plus disease, urgent treatment is needed.

## Documenting and communicating findings and management decisions

It is very important that accurate records are kept for all babies who have been screened for ROP. This will help to ensure that babies are screened at the right time and that follow-up screening is done as and when needed. If a baby is not screened when they should have been, they are more likely to become visually impaired or blind. At each screening, document all findings for both eyes (immature retinal vessels, stage, zone, plus disease, aggressive posterior, ROP is regressing). Note whether treatment or further screening is needed, and when.

Finally, ensure that all information is shared with the neonatal team and the parents. ROP is a complex disease with long-term consequences and requires a team effort (see pp. 60–61).

